# Better caregiver mastery is associated with less anxiety in individuals with cognitive impairment

**DOI:** 10.1186/s12912-023-01471-x

**Published:** 2023-09-07

**Authors:** Yeji Hwang, Miranda V. McPhillips, Liming Huang, G. Adriana Perez, Nancy A. Hodgson

**Affiliations:** 1 College of Nursing and Research Institute of Nursing Science, Seoul National University, 103 Daehak-ro, Jongno-gu, Seoul, 03080 Korea; 2 University of Pennsylvania School of Nursing, Philadelphia, PA USA

**Keywords:** Caregivers, Cognitive dysfunction, Behavioral and psychological symptoms of Dementia, Anxiety, Self-efficacy

## Abstract

**Background:**

When caregivers have a high level of caregiver mastery, their care recipients with cognitive impairment have less behavioral health problems. However, the relationship between caregiver mastery and anxiety among care recipients over time is unknown. Therefore, this study was conducted to examine that better caregiver mastery is associated with less anxiety in individuals with cognitive impairment over time.

**Methods:**

A secondary data analysis was conducted using the Healthy Patterns Clinical Trial (NCT03682185) dataset and guided by Factors Associated with Behavioral and Psychological Symptoms of Dementia conceptual framework. This study included 154 dyads of individuals with cognitive impairment and their caregivers. Multiple linear regression analyses were performed on changes in anxiety. Model 1 included variables at the level of neurodegeneration (i.e., cognitive impairment and age). Model 2 added patient factors (i.e., sleep problems and depression) with the Model 1. Finally, Model 3 included caregiver factor (i.e., caregiver mastery) with the Model 2 to examine how changes in caregiver mastery influence changes in anxiety of care recipients.

**Results:**

Model 3 was statistically significant; after controlling for variables at the level of neurodegeneration associated with cognitive impairment and patient factors, improvement of caregiver mastery over time (β =-0.230, p = 0.015) was related to decreased anxiety over time (R^2^ = 0.1099).

**Conclusions:**

Caregivers with high caregiver mastery may have better knowledge on how to care for their loved ones and how to manage their neuropsychiatric symptoms. Therefore, improving the level of caregiver mastery by providing psychoeducational programs and resources that family caregivers need will help reduce the frequency of anxiety in individuals with cognitive impairment.

## Background

The number of individuals with cognitive impairment, including mild cognitive impairment and dementia, is constantly increasing as the baby boomer generation continues to aging [1]. There are 11 million family and unpaid caregivers in the United States [2]; more than one third of all family caregivers who provide care for older adults are providing care for individuals with cognitive impairment [3]. Given the nature of cognitive impairment and loss of ability to complete daily tasks, family caregivers are instrumental in the daily lives of individuals with cognitive impairment, especially for those living at home [2].

Neuropsychiatric symptoms, or behavioral and psychological symptoms are common in individuals with cognitive impairment and can cause a great deal of distress for both caregivers and individuals with cognitive impairment [2]. Anxiety is one common neuropsychiatric symptom present in individuals with cognitive impairment, and it is reported that up to 71% of individuals with cognitive impairment experience anxiety [4]. Anxiety is related to poor quality of life for individuals with cognitive impairment [5, 6] and it also increases family caregivers’ burden [7, 8].

Managing neuropsychiatric symptoms is a key element when caring for individuals with cognitive impairment [2, 9]. While underlying causes for these symptoms are unknown, according to previous literature, patient factors, caregiver factors, and environmental factors can trigger or exacerbate the behavioral and psychological symptoms [10, 11]. In particular, caregiver factors such as caregiver mastery can influence behavioral and psychological symptoms of individuals with cognitive impairment [10].

The definition of caregiver mastery is a positive view of one’s ability and ongoing behavior during the caregiving process [12]. Mastery reflects self-perception of one’s efficacy in caregiving or confidence [12]. Previous studies have reported beneficial influence of caregiver’s positive self-perception in caregiving on care recipients’ health outcomes [13–16]. For example, high caregiver mastery in their ability to provide care was positively associated with care-recipient’s self-reported health [14]. Furthermore, in another study, poor family mastery was related to greater depression and anxiety among children with epilepsy [16].

**Fig. 1:**
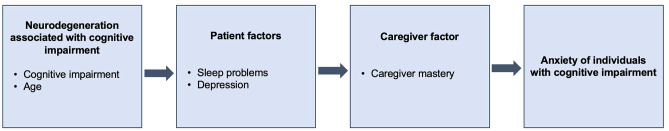
Conceptual Framework of the Study

Similar relationships exist in those caring for individuals with cognitive impairment [13, 15]. Poor caregiver mastery was related to increased frequency and severity of neuropsychiatric symptoms of individuals with cognitive impairment [15]. Better caregiver mastery was related to a decreased chance of developing anxiety among individuals with cognitive impairment [13]. These results suggest that caregiver mastery may contribute to decreasing neuropsychiatric symptoms such as anxiety in individuals with cognitive impairment but are limited by the cross sectional design [13, 15]. The longitudinal relationship between caregiver mastery and anxiety among individuals with cognitive impairment remains unknown. Therefore, the objective of this study was to examine the relationship between caregiver mastery and care recipients’ anxiety over time. The conceptual model of Factors Associated with Behavioral and Psychological Symptoms of Dementia [10, 11] guided this study. According to this model, neuropsychiatric symptoms of individuals with cognitive impairment can be triggered by neurodegeneration associated with cognitive impairment, patient-related factors such as unmet needs (e.g., sleep impairments, personal characteristics), and caregiver-related factors (e.g., caregiver mastery) [11]. We adopted the Factors Associated with Behavioral and Psychological Symptoms of Dementia model to design a conceptual framework for this study (Fig. 1). The three categories, neurodegeneration associated with cognitive impairment, patient factors, and caregiver factor, were from the original model. For neurodegeneration associated with cognitive impairment, we included age and cognitive impairment stage based on the original model [10, 11], their relationship to anxiety [17, 18], and availability of variables from the parent study [19]. For patient-related factors, sleep problems and depression were included based on the original model [10, 11], their relationships to anxiety [13, 20–22], and availability of variables from the parent study [19]. For caregiver-related factor, we included caregiver mastery as this was our key research question. We hypothesized that improvement in caregiver mastery over time would be related to reduced anxiety in individuals with cognitive impairment, after controlling for patient factors.

## Methods

### Study Design and setting

This was a secondary data analysis using data from Healthy Patterns Clinical Trial, a randomized control trial of a home-based activity intervention designed to improve circadian rhythm disorders and quality of life in community-residing older adults who have cognitive impairment (NCT03682185) [19]. This 1-month program targeted individuals with cognitive impairment and their family caregivers and had an experimental group and an attention control group [19]. In this current study, we used the pretest (T1) and posttest (T2) of the parent study to examine the relationship between changes in caregiver mastery and changes in anxiety among individuals with cognitive impairment.

### Participants and data Collection

The parent study enrolled participants who: (1) were over 55 years old, (2) could speak English or Spanish, (3) lived in the greater Philadelphia area, (4) could tolerate wrist actigraphy, (5) were diagnosed with probable dementia based on standard assessments and diagnostic criteria, (6) had caregiver-reported symptoms of sleep-wake disturbances and circadian rhythm disorders, and (7) were taking a stable dose of psychotropic medication for 90 days if prescribed [19]. In the parent study, caregivers were family members or friends who informally cared for the participants with cognitive impairment. The parent study collected the data between February 2017 and February 2020. All participants provided written informed consents for the study. Additional information on the parent study participants and data collection methods can be found elsewhere [19]. For the data collection at the pretest (T1) and posttest (T2), the research staff visited the participants’ home and collected the data using surveys. In this study, a total of 154 dyads who provided information on anxiety frequency and severity (dependent variable) both at T1 and T2 were analyzed. Although this study was a secondary data analysis of an existing data, we conducted a power analysis using G Power 3.1 software to ensure that the sample size is sufficient to answer our research question. With the medium effect size (Cohen’s f^2^ = 0.15), power of 0.80, probability of 0.05, and five predictors, the required sample size was 92. Therefore, the sample size of this study was sufficient.

### Measures

#### Demographic Information

Demographic information was collected for both individuals with cognitive impairment and their caregivers at T1. For individuals with cognitive impairment, age, sex, race/ethnicity, and education were collected. For caregivers of individuals with cognitive impairment, information on age and sex was collected.

#### Neurodegeneration associated with cognitive impairment

Neurodegeneration level factors included age and cognitive status of individuals with cognitive impairment.

#### Patient factors

Cognitive status was measured with the Clinical Dementia Rating (0.5 = very mild dementia; 1 = mild; 2 = moderate; 3 = severe) [23]. Patient level factors included changes in sleep impairment and changes in depression. Sleep impairment was measured with the PROMIS Sleep Related Impairment Short Form [24, 25]. In this study, the reliability of this 8-item scale was 0.82 for T1 and 0.88 for T2. Caregivers rated symptoms of sleep impairment of the care recipients on a 5-point Likert scale and the total score was standardized [24, 25]. Higher scores indicated more sleep impairment; change in sleep impairment was calculated by subtracting the T1 score from T2 score (T2-T1). Negative values indicated improvement in sleep impairment over time.

Depression was measured with Patient Health Questionnaire-9 [26]. This 9-item was on 4-point Likert Scale to ask caregivers to describe their care recipients’ depressive symptoms within the past 2 weeks. Higher scores indicated more depression. The reliability of the scale was 0.85 for both T1 and T2. Change in depression (T2-T1) was included as a covariate in this study, with negative scores indicating improvement in depression over time.

#### Caregiver factor

Caregiver mastery was considered as a caregiver factor. Caregiver mastery was measured with the Caregiving Mastery Index [12, 27]. The Caregiving Mastery Index is a 6-item scale that asks caregivers to evaluate their ability to care for their care recipients, ranging from never (1) to Always (5). The total possible score ranges from 6 to 30 and higher scores indicate high levels of caregiver mastery. Caregiver mastery was measured twice, at T1 and T2, and the reliability of the scale was acceptable in this study with Cronbach’s alpha value of 0.68 for T1 and 0.66 for T2. The change score between the two time points was calculated by subtracting T1 value from T2 (T2-T1). Positive scores indicated that caregiver mastery improved over time.

#### Anxiety of Individuals with Cognitive Impairment

The dependent variable of this study was change in anxiety frequency and changes in anxiety severity. Anxiety among individuals with cognitive impairment was measured with one item of the Neuropsychiatric Inventory [28–30] by asking caregivers: “Is the patient very nervous, worried, or frightened for no apparent reason? Does he/she seem very tense or fidgety? Is the patient afraid to be apart from you?” The NPI was developed to measure symptom states present in the past few weeks. In our study, participants first provided yes or no to the anxiety question. Then, anxiety frequency and anxiety severity were measured. If anxiety was not present, those were coded as 0 for both anxiety frequency and severity. Responses for anxiety frequency were scored with a 5-point Likert scale: No anxiety (0), rarely-less than once a week (1), sometimes-about once per week (2), often-several times per week but less than everyday (3), and very often-essentially continuously present (4). Anxiety severity was scored with a 4-point Likert scale: No anxiety (0), mild (1), moderate (2), severe (3). Both anxiety frequency and anxiety severity of individuals with cognitive impairment were measured at T1 and T2. For the purpose of this study, change in anxiety frequency was calculated by subtracting anxiety frequency at T1 from anxiety frequency at T2 (T2-T1). Positive values indicated increase in frequency over time. Similarly, change in anxiety severity was calculated by subtracting anxiety severity at T1 from anxiety severity at T2 (T2-T1), and positive values indicate increase in severity over time.

### Data Analysis

Descriptive analyses in this study included means, standard deviations, and percentages. In order to ensure there were no differences based on the group assignment before conducting the main analyses, we used independent t-tests to examine differences in all variables based on group assignment. Next, stepwise multiple regression analyses were performed on changes in anxiety frequency following the conceptual framework of the study. Model 1 included variables at the level of neurodegeneration associated with cognitive impairment (i.e., cognitive impairment and age). Model 2 added patient factors (i.e., sleep problems and depression) in addition to Model 1. Finally, Model 3 included caregiver factor (i.e., caregiver master) in addition to Model 2 to examine how changes in caregiver mastery influence changes in anxiety of care recipients. We used listwise deletion and only the complete cases were used for the final regression model [31]. Multicollinearity was checked using variance inflation factors. Stata BE 17 [32] was used for all statistical analyses. The level of statistical significance was set at alpha = 0.05. This secondary data analysis used the STROBE checklist for reporting cohort studies. For the regression models, we used listwise deletion methods [31], and 39 dyads (25%) dropped out because of missing data. We compared all variables used in the models between the complete cases and those with missing data. There were no statistical differences between two groups in terms of age, cognitive impairment, changes in sleep, changes in depression, changes in caregiver mastery, changes in anxiety frequency, and changes in anxiety severity. Multicollinearity was not detected with all variance inflation factors (VIF) were less than 2.

## Results

Table 1 describes the characteristics of the individuals with cognitive impairment and their family caregivers (N = 154). The mean age of the participants was 73.26 ± 8.41 years. The majority of study participants were Black or African Americans, had very mild dementia, and had high school education or less. The mean age of the family caregivers was 55.8 ± 13.8 years and 83.4% of the caregivers were female.

**Table 1:** Characteristics of the study participants (N = 154)†

	Variables	Baseline (T1)		Follow-up (T2)		Changes in variables (T2-T1)	
		n(%) or M ± SD	Min, Max	n(%) or M ± SD	Min, Max	n(%) or M ± SD	Min, Max
Persons living with cognitive impairment	Age	73.26 ± 8.41	55, 98				
	Sex						
	Female	105 (66.5)					
	Male	52 (33.8)					
	Race/Ethnicity						
	Black or African American	96 (63.2)					
	Hispanic/Latino	37 (24.3)					
	White	19 (12.5)					
	Clinical Dementia Rating (CDR)	0.73 ± 0.50	0.5, 3				
	0.5 (Very Mild Dementia)	111 (73.0)					
	1.0 (Mild Dementia)	29 (19.1)					
	2.0 (Moderate Dementia)	9 (5.9)					
	3.0 (Severe Dementia)	3 (2.0)					
	Education						
	Less than 9 years	27 (18.1)					
	High School	57 (38.3)					
	Some College	35 (23.5)					
	College	22 (14.8)					
	Graduate Degree	8 (5.4)					
	Sleep Impairment	48.35 ± 9.74	30, 71.9	46.84 ± 9.26	30, 75	-1.32 ± 6.55	-18.5, 20.9
	Depression	5.41 ± 5.21	0, 27	4.31 ± 4.81	0, 24	-0.83 ± 2.74	-9, 8
	Anxiety Frequency	0.77 ± 1.27	0, 4	0.56 ± 1.10	0, 4	-0.21 ± 1.00	-4, 3
	Anxiety Severity	0.59 ± 0.97	0, 3	0.44 ± 0.79	0, 3	-0.16 ± 0.79	-3, 2
Caregivers	Age	55.94 ± 13.83	22, 90				
	Sex						
	Female	129 (84.3)					
	Male	24 (15.7)					
	Caregiver Mastery	23.57 ± 3.93	15, 30	24.17 ± 3.73	14, 30	0.41 ± 2.92	-8, 9

^†^Notes: For the changes in sleep impairment (T2-T1), the greater the negative score indicates improvement of sleep impairment over time. The greater the negative scores of the changes in depression (T2-T1) indicates improvement of depressive symptoms over time. The greater score in changes in caregiver mastery indicate that caregiver mastery improved over time. For the changes in anxiety frequency (T2-T1), the greater the negative scores indicate that anxiety frequency decreased over time. For changes in anxiety severity (T2-T1), the greater the negative scores indicate that anxiety severity improved over time

For the 1-month period from the pretest and posttest, the mean change of sleep impairment and depression was − 1.32 ± 6.55 (p = 0.020) and − 0.83 ± 2.74 (p = 0.008), respectively, indicating that sleep impairment and depression decreased over time. The mean change of caregiver mastery among family caregivers was 0.41 ± 2.92 (p = 0.118) indicating that caregiver mastery improved over time though it was not statistically significant. The mean change of anxiety frequency was − 0.21 ± 1.00 (p = 0.011) indicating that anxiety frequency decreased over time. The mean change of anxiety severity was − 0.16 ± 0.79 (p = 0.012) indicating that anxiety severity improved over time.

Independent t-tests were conducted to compare the means for all variables between the experimental and control groups (Table 2). There were no statistical differences in all variables including changes in anxiety frequency, anxiety severity, and caregiver mastery score. In other words, in this secondary data analysis, there were no differences based on group assignment. Therefore, we did not include the variable of group assignment in following regression models.

**Table 2:** Difference of the Variables by the Group (N = 154)

		Healthy Patterns Intervention Group (n = 71)	Control Group (n = 83)	t	df	p
		M ± SD	M ± SD			
Neurodegeneration associated with cognitive impairment	Cognitive impairment (T1)	0.80 ± 0.64	0.67 ± 0.34	-1.544	103.287^†^	0.126
	Age (T1)	73.54 ± 8.50	73.02 ± 8.38	-0.374	151	0.709
Patient factors	Changes in sleep impairment (T2-T1)	-2.10 ± 6.34	-0.70 ± 6.68	1.236	134	0.219
	Changes in depression (T2-T1)	-1.21 ± 2.53	-0.52 ± 2.53	1.420	126	0.158
Caregiver factor	Changes in caregiver mastery (T2-T1)	0.88 ± 1.97	-0.02 ± 3.51	-1.811	107.622^†^	0.073
Anxiety of individuals with cognitive impairment	Changes in anxiety frequency (T2-T1)	-0.23 ± 0.72	-0.19 ± 1.19	0.208	127.534^†^	0.835

Notes: For the changes in sleep impairment (T2-T1), the greater the negative score indicates improvement of sleep impairment over time. The greater the negative scores of the changes in depression (T2-T1) indicates improvement of depressive symptoms over time

^†^ Satterthwaite’s degrees of freedom with the unequal variances

Table 3 shows the linear regression analysis models on change in anxiety frequency. Model 1 included the variables at the neurodegeneration associated with cognitive impairment (R^2^ = 0.0062, F = 0.46, p = 0.631). Next, Model 2 included patient factors in addition to Model 1 (R^2^ = 0.0583, F = 1.84, p = 0.125). Finally, Model 3 included caregiver factor in addition to Model 2 (R^2^ = 0.1099, F = 2.74, p = 0.023). Model 3 was statistically significant and indicated that after controlling for variables at the level of neurodegeneration associated with cognitive impairment and the level of patient factors, improvement of caregiver mastery over time (β=-0.230, SE = 0.033, t=-2.48, p = 0.015) was related to decreased anxiety frequency over time.

**Table 3:** Multivariable linear regression models on changes in anxiety frequency (n = 115)

		Model 1				Model 2				Model 3			
Level	Variables	β	SE	t	p	β	SE	t	p	β	SE	t	p
Neurodegeneration associated with cognitive impairment	Age	0.032	0.010	0.38	0.708	-0.061	0.111	-0.67	0.504	-0.056	0.012	-0.61	0.546
	Clinical Dementia Rating (CDR)	0.066	0.169	0.78	0.436	0.095	0.206	1.05	0.297	0.056	0.215	0.59	0.553
Patient factors	Changes in sleep impairment (T2-T1)					0.230	0.014	2.52	0.013	0.228	0.015	2.45	**0.016**
	Changes in depression (T2-T1)					0.009	0.033	0.10	0.924	-0.017	0.035	-0.19	0.853
Caregiver factor	Changes in caregiver mastery (T2-T1)									-0.230	0.033	-2.48	**0.015**
	Constant		0.724	-0.81	0.420		0.810	0.32	0.750		0.847	0.36	0.722
		R^2^ = 0.0062, F = 0.46, p = 0.631				R^2^ = 0.0583, F = 1.84, p = 0.125				R^2^ = 0.1099, F = 2.74, p = 0.023			

Similarly, we conducted the linear regression analyses on changes in anxiety severity based on the conceptual framework of the study (Table 4). However, none of the models were significant (Model 1: R^2^ = 0.0057, F = 0.42, p = 0.657; Model 2: R^2^ = 0.070, F = 2.25, p = 0.068; Model 3: R^2^ = 0.084, F = 2.01, p = 0.083).

**Table 4:** Multivariable linear regression models on changes in anxiety severity (n = 115)

		Model 1				Model 2				Model 3			
Level	Variables	β	SE	t	p	β	SE	t	p	β	SE	t	p
Neurodegeneration associated with cognitive impairment	Age	0.061	0.008	0.73	0.467	-0.033	0.008	-0.36	0.718	-0.030	0.009	-0.32	0.753
	Clinical Dementia Rating (CDR)	0.032	0.128	0.39	0.700	0.052	0.153	0.58	0.562	0.045	0.164	0.47	0.636
Patient factors	Changes in sleep impairment (T2-T1)					0.257	0.011	2.83	0.005	0.268	0.011	2.82	0.006
	Changes in depression (T2-T1)					0.039	0.025	0.43	0.669	0.026	0.026	0.28	0.780
Caregiver factor	Changes in caregiver mastery (T2-T1)									-0.077	0.026	-0.82	0.416
	Constant		0.550	-1.10	0.275		0.604	0.10	0.919		0.646	0.08	0.939
		R^2^ = 0.0057, F = 0.42, p = 0.657				R^2^ = 0.070, F = 2.25, p = 0.068				R^2^ = 0.084, F = 2.01, p = 0.083			

## Discussion

The purpose of this study was to examine the relationship between caregiver mastery and care recipients’ anxiety over time. Based on the conceptual framework, we tested whether a caregiver factor can influence anxiety of individuals with cognitive impairment, after controlling for neurodegenerative associated with cognitive impairment and patient factors. Improvement in caregiver mastery over time was related to reduced anxiety frequency among individuals with cognitive impairment.

Our study finding is similar to previous studies which examined the relationship between caregiver mastery and neuropsychiatric symptoms of the care recipients with cognitive impairment [13, 15]. Low level of caregivers’ self-efficacy was related to neuropsychiatric symptoms of individuals with cognitive impairment [15]. A higher level of caregiver mastery was also related to lower odds of anxiety presentation in individuals with cognitive impairment [13]. The results of this study strengthen the argument that caregivers’ characteristics can influence care recipients’ health and wellbeing [10]. Although caregiver level factors are modifiable factors associated with anxiety in individuals with cognitive impairment, a limited number of research are available which examined the relationship between caregiver level factors and anxiety of the care recipients [33].

In the current study, we found that when the level of caregiver mastery is increased, care recipients less frequently experience anxiety. Because mastery is a self-perception of one’s ability to do something, this can be influenced from many other conditions and change [12]. There is a possibility that caregivers with high caregiver mastery may have better knowledge on how to care for their loved ones and how to manage their neuropsychiatric symptoms. Kales et al. (2015) explained that caregivers’ psychological wellbeing can trigger or exacerbate symptoms of individuals with cognitive impairment because of their essential roles in dementia care [11]. In other words, caregivers’ stress, depression, or low caregiver mastery, can interact with other environmental factors and worsen the symptoms [10, 11]. Caregivers’ high confidence in dementia care might have been related to better psychological wellbeing which positively influenced anxiety of the care recipients [10, 13].

The results of this study are aligned with previous literature which explained caregiver factors such as caregiver mastery could influence the behavioral and psychological symptoms of the care recipients with cognitive impairment [10, 11]. Given that a high level of caregiver mastery can lessen the frequency of anxiety in individuals with cognitive impairment, future research will need to focus on improving confidence or mastery of family caregivers of individuals with cognitive impairment.

In this study, the regression model on anxiety frequency was significant while the model on anxiety severity was not significant. This is in line with a previous study which showed that caregiver characteristics can influence presence of anxiety in persons whom they are caring for [13]. Further research is needed on how caregiver mastery is related to anxiety frequency and anxiety severity of the care recipients.

Caregiver mastery is not only important for the wellbeing of the care recipients but also the wellbeing of caregivers themselves. Poor caregiver mastery is associated with greater depressive symptoms among family caregivers of people with chronic illness [34, 35]. High level of caregiver mastery also has protective effect against acute stress among caregivers [36, 37]. Even when caregivers felt high levels of caregiver burden, high levels of mastery reduced the risk of death among caregivers [38]. Because caregiver mastery has positive effects both on caregiver themselves and their care recipients, nurses need to evaluate caregivers’ level of caregiver mastery, and if needed, provide resources that are helpful in dementia care.

Psychoeducational intervention programs which aimed to develop caregivers’ skills have increased in recent years, and these interventions have been shown to be effective in improving caregiver mastery [39–41]. In addition, another type of intervention program which provided tailored activities for individuals living with dementia based on their capabilities and interests also reduced frequency of behavioral problems of individuals with dementia and improved caregiver mastery over time [42]. Other types of intervention programs such as providing education and resources to dementia caregivers [43] or training caregivers on how to assess neuropsychiatric symptoms and manage neuropsychiatric symptoms of individuals with cognitive impairment [10, 44] were also effective in improving caregivers’ confidence in care. Many families lack information and resources that they need to take care of persons living with cognitive impairment [45]. Therefore, improving the level of caregiver mastery by providing psychoeducational programs and resources that family caregivers need will help reduce the frequency of anxiety in individuals with cognitive impairment.

When nurses conduct assessments for individuals with cognitive impairment, we suggest that they also evaluate caregivers’ mastery and confidence in care, because they can influence anxiety of the care recipients. The wellbeing of caregivers is important for both the caregivers themselves and for the loved ones for whom they provide care. Therefore, efforts are needed to improve caregiver mastery by providing education and resources for caregivers so that anxiety level of the care recipients can be decreased. Implementing evidence-based nursing intervention programs in real-life settings aimed at improving caregiver mastery among dementia caregivers will benefit both caregivers and care recipients.

Strengths of this study include the longitudinal design and the unique composition of the sample. To the best of our knowledge, the parent study is one of the largest samples of Black/African American individuals in the community with cognitive impairment and their identified caregivers. It is important to include diverse population in dementia research because racial disparities are reported in dementia care [46, 47]. This study also filled the gap in the literature on how caregivers could influence anxiety symptoms of individuals with cognitive impairment. In addition, the use of a continuous variable of anxiety frequency in this study is also an advantage because previous studies focused only on the presence of anxiety.

There are a few limitations as well. First, since this is a secondary data analysis, we could not control for other covariates that may have influenced caregiver mastery or anxiety, such as relationship factors between the dyads, hours caregiving per day or anxiety status of the caregiver. Second, we used proxy-reported anxiety given that the sample has cognitive impairment. The caregivers with higher mastery may have been more confident which could have subsequently influenced their rating of caregiver anxiety. Additionally, anxiety, as measured by the Neuropsychiatric Inventory only measures state anxiety only, asking about symptoms in the past few weeks. Because both trait and state anxiety are important, future studies should consider both aspects. Lastly, although the time between the pretest (T1) and posttest (T2) was one month for each dyad, because the parent study collected data for three years, each dyad participated in this study at different timepoint during the three-year period. That said, data collection commenced prior to the start of Covid-19.

## Conclusions

This study found that increased level of caregiver mastery was associated with reduced anxiety frequency among individuals with cognitive impairment. The results of this study strengthen the argument that caregivers’ characteristics can influence health and wellbeing of care recipients living with cognitive impairment. Nursing interventions to improve caregiver mastery are needed as they may alleviate anxiety symptoms in individuals with cognitive impairment.

## Data Availability

The datasets used and/or analyzed during the current study are available from Dr. Nancy Hodgson on reasonable request.
